# Macaronesian Plants as Promising Biopesticides against the Crop Pest *Ceratitis capitata*

**DOI:** 10.3390/plants12244122

**Published:** 2023-12-10

**Authors:** Wilson R. Tavares, Ignacio A. Jiménez, Luísa Oliveira, Maria Kuhtinskaja, Merike Vaher, José S. Rosa, Ana M. L. Seca, Isabel L. Bazzocchi, Maria do Carmo Barreto

**Affiliations:** 1 Centre for Ecology, Evolution and Environmental Changes (cE3c), Azorean Biodiversity Group & Global Change and Sustainability Institute (CHANGE), Faculty of Sciences and Technology, University of the Azores, 9501-321 Ponta Delgada, Portugal; wilson.r.tavares@uac.pt (W.R.T.); ana.ml.seca@uac.pt (A.M.L.S.); 2 Instituto Universitario de Bio-Orgánica Antonio González, Departamento de Química Orgánica, Universidad de La Laguna, Avenida Astrofísico Francisco Sánchez 2, 38206 La Laguna, Spain; 3 CBA—Biotechnology Centre of Azores, Faculty of Sciences and Technology, University of the Azores, 9501-321 Ponta Delgada, Portugal; maria.lm.oliveira@uac.pt (L.O.);; 4 Department of Chemistry and Biotechnology, Tallinn University of Technology, Akadeemia tee 15, 12618 Tallinn, Estonia; maria.kuhtinskaja@taltech.ee (M.K.); merike.vaher@taltech.ee (M.V.); 5 Associated Laboratory for Green Chemistry (LAQV) of the Network of Chemistry and Technology (REQUIMTE), Department of Chemistry, University of Aveiro, 3810-193 Aveiro, Portugal

**Keywords:** *Ceratitis capitata*, pest control, Macaronesian species, ethanolic extracts, *Hedychium gardnerianum*, *Salvia canariensis*, myricetin, quercetin, HPLC-MS/MS

## Abstract

*Ceratitis capitata* is responsible for significant economic losses in the fruit production industry, and the market lacks biopesticides that are effective but also cheaper and less contaminating, with fewer negative impacts on the environment. In this regard, the present study suggests as potential options ethanolic extracts from several Macaronesian plants, which inhibit the oviposition and are toxic to *C. capitata*, and whose preparation involve a non-toxic solvent (i.e., ethanol), low energy expenditure and cheap apparatus (i.e., maceration at room temperature). Among the evaluated species, the extracts of *Hedychium gardnerianum*, *Cistus symphytifolius* and *Salvia canariensis* are the most active (50 mg/mL), revealing an increase in *C. capitata* adults’ mortality from 21.15% to 27.41% after 72 h, a value statistically identical to azadirachtin (25.93%) at the recommended concentration (0.88 mg/mL). Considering the quantity and biomass available to prepare a biopesticide in the future, and the level of activity, the ethanolic extract of *H. gardnerianum* was fractionated and each fraction tested. The water fraction at 50 mg/mL proved to be more effective than the original extract, both in terms of mortality (57.69%), with LT_50_ = 72.5 h, and oviposition deterrence (83.43%), values statistically higher than those obtained by azadirachtin at 0.88 mg/mL. Analysis of this fraction by HPLC-MS/MS showed that it is mainly composed of glycosylated derivatives of quercetin and myricetin in addition to some triterpenes. These findings highlight some Macaronesian species, and in particular, the more polar fraction of *H. gardnerianum* ethanolic extract, as promising and ecological alternatives to conventional insecticides, for use in the integrated management of the *C. capitata* pest.

## 1. Introduction

*Ceratitis capitata*, the Mediterranean fruit fly, also known as medfly, is a polyphagous species that has spread from its supposed origin in Africa to several regions of the world and is considered a major problem for the fruit market since it is able to infest the fruits of over 300 species of plants, causing significant economic losses to agricultural communities [1,2,3,4]. *Ceratitis capitata* can act as a potential vector for *Erwinia amylovora*, which causes fire blight in apple and pear orchards [5]. The development of *C. capitata* larvae in the fruit tissue is mediated by bacterial decay; thus, the larvae, often derived from the oviposition from different females and therefore with different microbial populations, play a major role in spreading the bacteria in the fruits, increasing the damage throughout the orchards [6].

The main strategy in controlling this pest is the use of organophosphate insecticides, especially malathion bait sprays, causing resistance in the insects after continuous application [7] and toxicity to mammals focused on their central nervous system, blood and various organs [8]. Replacing synthetic pesticides with plant-based natural compounds that have lower toxicity is not only safer for consumers but also essential to reduce the environmental impact, namely decreasing the contamination of soil and water resources by xenobiotics, while continuing to effectively protect cultures and stored crops from pests [9].

The use of plants to protect crops against insect pests has been practiced since ancient civilizations, and it is still used by farmers around the world currently in the form of extracts, companion plants or just the harvested plant [10,11,12]. In recent years, commercial botanical insecticides have started to emerge due to the isolation of pure compounds with insecticidal action from several plants, such as pyrethrins (isolated from *Tanacetum cinerariifolium* Sch.Bip.) or azadirachtin (isolated from *Azadirachta indica* A. Juss.), which are two cases of successful natural insecticides [13]. Regarding the combating of *C. capitata*, there have been several studies on the potential of essential oils for its control, namely from *Tagetes* L. species [14], *Laurus azorica* (Seub.) Franco [15], *Lavandula dentata* L. [16] and *Lavandula stoechas* L. [16]. However, it is important to develop methods to control this pest that are not only efficient and environmentally safe but also simpler to prepare involve non-toxic solvents, have low energy expenditure and need inexpensive apparatus [17]. An example is using aqueous and ethanolic extracts, prepared from available biomass, based on simple processes that can be easier to prepare and cheaper for farmers, such as maceration at room temperature [18,19]. This strategy to develop biopesticides will contribute to the economic viability and sustainability of agriculture [17].

Taking this into account, ethanolic extracts from plants collected in the Azores and the Canary Islands were prepared and their biopesticide effects against *C. capitata* evaluated. Following a bioguided strategy, the most promising extract was fractionated. The chemical composition of the fraction that presented the highest insecticide action against *C. capitata* was analyzed using the HPLC-PDA-MS/MS technique. In addition, the suggested methodology for preparing the extracts is intended to ensure the easy transfer of knowhow to farmers, allowing them to prepare the biopesticides to be used by themselves.

## 2. Results

### 2.1. The Extraction Yields

Ethanolic extracts were prepared from seven plants collected in the Azores and the Canary Islands. The extraction yields are shown in Table 1.

### 2.2. Ceratitis capitata Adults’ Mortality after Contact with Treated Artificial Fruits

The mortality effect of ethanolic extracts from *Argyranthemum frutescens*, *Cistus symphytifolius*, *Laurus azorica*, *Maytenus canariensis*, *Salvia canariensis* and *Withania aristata* leaves and *Hedychium gardnerianum* stems and leaves, against the fruit fly *C. capitata*, was evaluated at 24, 48 and 72 h after treatment of artificial fruits (Table 2), prepared as described in the Materials and Methods section.

Time–response data obtained by the contact toxicity assay allowed determination of the lethal time (LT_50_ and LT_90_), i.e., the number of hours required to kill adult insects (Table 3). The *Hedychium gardnerianum* water fraction exhibits a LT_50_ value (72.54 h) around the assay time (72 h), and lower than the *n*-hexane fraction (95.29 h).

### 2.3. Oviposition Deterrent Activity (OD)

All the ethanolic extracts and the three fractions prepared were evaluated on their capacity to inhibit the oviposition and the obtained results are presented in Table 4.

### 2.4. Phytochemical Analysis

**Figure 1:**
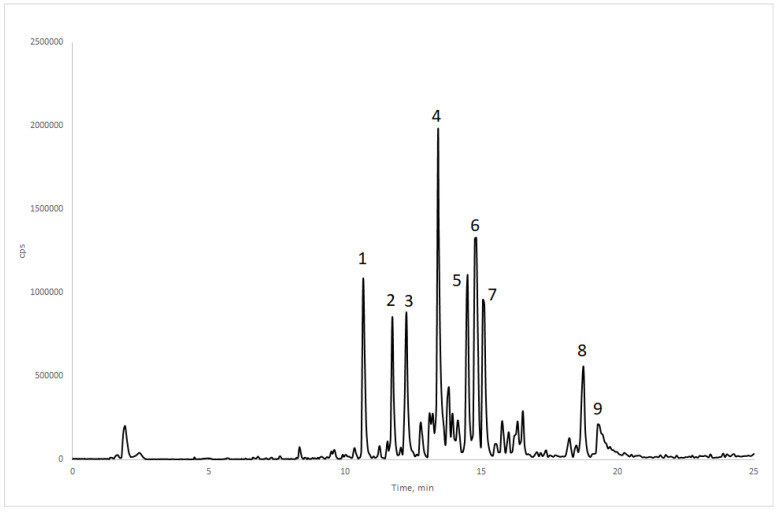
Total ion current (TIC) chromatogram of the water fraction (WF) from *Hedychium gardnerianum* ethanolic extract (EE). The peaks **1**–**9** are labelled according to the compounds in Table 5.

The water fraction (WF) of the ethanolic extract from *H. gardnerianum* presented the strongest activity against *C. capitata*, exhibiting high oviposition deterrent (83.43%) and mortality rate (57.69% at 72 h) activities and the lowest LT_50_ (72.5 h). Thus, this very polar fraction was analysed by HPLC-PDA-MS/MS to better understand its chemical profile (Figure 1).

The nine most abundant peaks (**1** to **9**) were subjected to characterization by MS/MS and, based on the fragmentation pattern analysis, they were putatively identified as shown in Table 5 (the compounds are labelled according to Figure 1).

Based on the MS deprotonated pseudomolecular ions and MS/MS product ions obtained for each peak (Table 5), it was possible to determine which family of natural compounds each one belongs to. Six of the nine peaks (**1**, **2**, **4, 5, 6** and **7**) belong to flavonol glycosides, exclusively derived from the quercetin (compounds **2**, **5** and **7**) and myricetin (compounds **1**, **4** and **6**) aglycones, characterized by the MS/MS product ions at *m*/*z* 301 and 317, respectively, after elimination of the sugar residues [20]. The predominance of the signals at *m*/*z* 301 and 317 (corresponding to the negative aglycone ion), instead of the more intense signals at *m*/*z* 300 and 316 (corresponding to the radical aglycone ion), shows the prevalence of flavonol-3-*O*-glycoside bonds over flavonol-7-*O*-glycoside bonds [21]. The neutral loss of 176 Da indicates the presence of a uronic acid unity, while losses of 162 Da and 146 Da mean there is the loss of a hexoside (glucose or galactose moiety) and a deoxyhexoside (rhamnose moiety), respectively, and finally, a loss of 308 Da shows the presence of a rutinoside or neohesperidoside moiety, in all cases linked to the aglycone by an *O*-glycosidic bond [22,23].

Compound **5** was identified by comparison with the standard quercetin–3-*O*-rutinoside (quercetin-3-*O*-(α-L-rhamnopyranosyl-(1→6)-β-D-glucopyranose) analyzed under the same conditions, with the MS signal at *m*/*z* 609 [M-H]^−^ and MS/MS product ion at *m*/*z* 301 [M-H-308]^−^ being characteristic of rutin.

The MS data indicate that compound **2** (deprotonated pseudomolecular ions at *m*/*z* 609 [M-H]^−^) is an isomer of compound **5**, both being aglycones of quercetin because of the signal at *m*/*z* 301 corresponding to the aglycone negative ion ${{[Y}_{0}]}^{-}$. However, the MS/MS also showed some differences. The structure proposed for compound **2** is based on what was observed on the MS/MS signals at *m*/*z* 463 and 447 corresponding to [M-H-146]^−^ and [M-H-162], respectively. These peaks show two alternative losses of sugar moieties, which indicates the presence of a quercetin-*O*-bisglycoside [24] and is confirmed by the literature data [21]. The observed losses show a quercetin aglycone linked to a hexose, which could be glucose or galactose, and to a deoxyhexose (rhamnose). Considering that in the *Hedychium* genus only glucosides and rhamnosides glycans are known [25], the most probable identification of compound **2** is quercetin-3-*O*-glucoside-7-*O*-rhamnoside.

Compound **7** is also a quercetin derivative because of the presence of the product ion at *m*/*z* 301. However, the MS quasimolecular ion at *m*/*z* 477 shows a quercetin-*O*-monoglycoside. The nature of the glycan can be deduced from the loss of 176 Da from [M-H]^−^, corresponding to loss of a uronic acid unity, identifying compound **7** as quercetin-3-*O*-glucuronide.

Compounds **1**, **4** and **6** are isomers, with the MS deprotonated pseudomolecular ions at *m*/*z* 625 and the MS/MS signal at *m*/*z* 317, revealing three compounds with myricetin as aglycone after losing a total of 308 Da. However, contrary to what was observed for compound **2**, the MS/MS spectra does not show both of the alternative product ions corresponding to [M-H-146]^−^ and [M-H-162]^−^, which means that compounds **1**, **4** and **6** are myricetin-3-*O*-di-glycosides with different sequences of glycoside parts [21], instead of myricetin-di-*O*-glycosides.

Compound **4** exhibits a very similar fragmentation pattern to compound **5**, characterized by a loss of 308 Da, showing the presence of a rutinose residue. However, as referred to above, compound **4** is a glycosylated derivative of myricetin, like compounds **1** and **6** (signal at *m*/*z* 317). Moreover, based on the literature data [26], compound **4** is tentatively identified as myricetin-3-*O*-rutinoside.

The MS/MS data of compound **6** exhibit a product ion at *m*/*z* 463 corresponding to [M-H-162]^−^, showing the hexoside as the terminal glycoside, while compound **1,** which exhibits a product ion at *m*/*z* 479 corresponding to [M-H-146]^−^, has a deoxyhexoside unit as the terminal residue. The product ions at *m*/*z* 463 and 479 are the precursors of the signal at *m*/*z* 317 corresponding to ${{[Y}_{0}]}^{-}$ after losses of the deoxyhexoside and hexoside units, directly linked to the aglycone, respectively. Thus, compounds **1** and **6** are tentatively identified as myricetin-3-*O*-rhamnosyl-glucoside and myricetin-3-*O*-glucosyl-rhamnoside, respectively.

The fragmentation pattern of compounds **8** and **9** includes losses of 60 (CH_3_COOH), 46 (HCOOH), 18 (H_2_O) and 15 Da [CH_3_], indicating the presence of acetyl, carboxyl, hydroxyl or methyl groups, respectively, and shows they belong to the triterpene family. Additionally, the MS and MS/MS analyses of compound **3** exhibit two sequential losses of 146 Da, at *m*/*z* 779 ([M-H-146]^−^) and 633 ([M-H-146-146]^−^), resulting from the cleavage of two deoxyl-hexoside units and indicating the presence of triterpene di-rhamnoside.

## 3. Discussion

Control of the Mediterranean fruit fly pest, *C. capitata*, is far from being achieved due to several factors, among them, the emergence of resistance to several insecticides in use, such as lambda-cyhalothrin and malathion, and the toxic effects of these pesticides [27]. The discovery and development of new insecticides is necessary to ensure sustainable control of this and other pests. Based on popular knowledge, seven Macaronesia plants that could serve as a source of biopesticides to combat the *C. capitata* pest were selected.

The percentage of adults’ mortality after contact with the artificial fruits treated with ethanolic extracts was initially low (less than 5 and 10%, respectively, at 24 and 48 h after treatment). In fact, at 24 h, no significant differences were detected among the extract treatments (*F* =1.717; df = 11; *p* = 0.079). Azadirachtin, at the recommended concentration after 24 h, also exhibits a mortality that is statistically no different from the control. For the other two periods (48 and 72 h), significant differences were detected among treatments (*F* = 2.704; df = 11; *p* = 0.028; and *F* = 2.739; df = 11; *p* = 0.004, respectively). In the three time periods used in the study, the mortality produced by *L. azorica* extract was low and was not significantly different from the values observed in the control group (*p* < 0.05), and the same was observed with ethanolic extracts from plants originating from the Canary Islands, except for *S. canariensis*. These results show that the biopesticide effect varies with the treatment’s duration and between samples tested. The same types of variations were observed in the literature [14,15], and the most likely explanation is related to the mechanism of action involved and the chemical composition of the samples. The highest mortality rates were observed at 72 h, for the ethanolic extracts of *S. canariensis*, *C. symphytifolius* and *H. gardnerianum*, with 27.41, 22.96 and 21.15%, respectively. Among these species, the first two are endemic to the Canary Islands, and therefore, the availability of biomass is much more limited, while the third is widely distributed on several islands in the Azores archipelago. *Hedychium gardnerianum* ethanolic extract was selected for further studies based on its high insecticide activity (mortality rate + OD) and abundance of biomass. This extract was fractionated obtaining the *n*-hexane (HF), ethyl acetate (EAF) and water (WF) fractions. Their activities were evaluated using the same procedure used for the extracts and the results obtained are also presented in Table 2.

The highest values of the adults’ mortality, significantly different from those observed in the azadirachtin group, were obtained with the water fraction (WF) after 48 h of treatment (*p* < 0.05), and WF and HF after 72 h of treatment (*p* < 0.05). The *n*-hexane and water fractions (HF and WF) exhibit a percentage of adults’ mortality after 72 h that is significantly higher than that exhibited by the extract before fractionation, while the ethyl acetate fraction is inactive. In fact, the water fraction reached a mortality rate greater than 50%, being statistically different (*p* < 0.05) from that shown by the positive control (i.e., azadirachtin mortality rate = 25.93%).

In general, most of the ethanolic extracts had a repulsive effect on the oviposition activity of *C. capitata* adults at the concentrations tested, with *A. frutescens* and *H. gardnerianum* exhibiting the highest capacity to inhibit oviposition (69.83 and 78.30%, respectively), better than azadirachtin (35.25%). Conversely, *W. aristata* caused an attractive activity (−44.41%) that was significantly different from what was observed in all other extracts, while *M. canariensis* did not seem to influence oviposition.

As observed from the mortality rate, the highest value of OD was observed in the presence of WF (83.43%), significantly higher than those observed in all the extracts and azadirachtin, followed by HF (78.70%), similar to *H. gardnerianum* EE’s activity (78.30%).

Looking to the *H. gardnerianum* and *A. frutescens* extracts, they were more efficient at deterring oviposition, although the differences between these species lack statistical significance. Although the ethanolic extracts evaluated did not exhibit insecticidal activities greater than those exhibited by the positive control azadirachtin, it is important to consider that they can be prepared by farmers in a simple and economical way.

Compared with the literature data, the effect of *L. azorica* essential oils against the medfly was already known [15]. However, it is difficult to compare the activity of the ethanolic extracts with the activity of the essential oils since the tests were carried out at different concentrations. In addition, it should be noted that the yield of essential oil extraction is notoriously lower than the yield of extracts prepared by maceration as described in the Materials and Methods section, as well as requiring specific extraction apparatus not easily available to farmers.

The results obtained by the water fraction of the ethanolic extract from *H. gardnerianum* stands out among the plants tested as being consistently more active against *C. capitata*, both in terms of mortality rates and oviposition deterrence.

Considering the chemical composition of this promising tested sample, flavonol glycosides and triterpene derivatives are the major constituents. The presence of flavonols and triterpenes in glycoside form is common in nature, allowing the solubility of specialized metabolites to be increased and their storage to be conducted in an inactive form [22]. Herein, only the quercetin and myricetin aglycones were identified, with this being the first time that these two aglycones have been reported in *H. gardnerianum*, while the correspondent glycoside is new in the *Hedychium* genus. Syringetin-3-rhamnoside, isolated from *Hedychium stenopetalum* G. Lodd., was the only flavonol glycoside previously identified in the genus [28]. However, the myricetin and quercetin *O*-glycosides are widespread and abundant in nature. In fact, all the flavonol glycosides tentatively identified in Table 5 have been described in the literature [26,29,30], some of which exhibit different pharmacological and pesticide activities. For example, quercetin–3-*O*-rutinoside (compound **5**) is described in the literature [31] as possessing a significant effect on larval development, pupal mortality and malformed adults of *Spodoptera litura*.

Considering the published literature [17,25], triterpenes are rare in *Hedychium* species, with lupeol being the only one characterized to date. The presence of saponin and other triterpene derivatives are reported here for the first time in the *Hedychium* genus. Consequently, the present work is innovative and contributes to filling the existing knowledge gap on the specialized metabolites biosynthesized by *Hedychium* species.

It should be noted that quercetin and myricetin were reported to interfere with the cytochrome P-450-dependent ecdysone 20-mono-oxygenase activity, important for molting and development in insect species such as *Aedes aegypti*, *Drosophila melanogaster* and *Manduca sexta,* affecting the viability of insect organisms, and being proposed by the authors of the paper as having potential as biopesticides [32]. Myricetin 3′-glucoside and myricetin hyperoside were also found by an in silico study to interfere with insect acetylcholinesterase receptors, actin, α-tubulin, arginine, kinase and histone receptor III subtypes, being capable of forming stable complexes with these proteins and inhibiting their activity [33]. The potential of quercetin in insect control is more frequently mentioned, namely as an oviposition deterrent of *Bactrocera dorsalis* and *B. correcta* [34], as an inhibitor of phenoloxidase from *Hyphantria cunea* (Lepidoptera: Arctiidae), a key enzyme in the development and immunity of insects [35], in the inhibition of the growth rate of cabbage looper *Trichoplusia ni* (Lepidoptera: Noctuidae) [36], and against the growth and development of the grasshopper *Oedaleus asiaticus* [37]. Saponins are also known to negatively interfere with insect metabolism, namely by a cytotoxic action due to permeation of the cell membrane [38]. Finally, triterpenes, such as azadirachtin, which was used here as a positive control, are known for their insecticidal [39,40] and oviposition deterrent activities [41].

Although similar in some respects, other recent works regarding the evaluation of extract-based biopesticide effects against *C. capitata* differ particularly in terms of the implemented methodology, concentration units and plant species used [18,19], thus making it difficult to compare results with the ones obtained in this study. Nevertheless, this work demonstrates the ability of ethanolic plant extracts to reduce oviposition while simultaneously causing mortality in *C. capitata*.

Since the best biopesticide effect was observed with the more polar fraction of the ethanolic extract of *H. gardnerianum*, future investigation should be focused on the biopesticide effect and quantitative chemical composition of the water extract against *C. capitata* and even against other pests. Additionally, the biopesticide effect of different polarity fractions of the ethanolic extract from cultivated *S. canariensis* must be studied.

## 4. Materials and Methods

### 4.1. Plant Material

Fresh leaves of *Argyranthemum frutescens, Cistus symphytifolius, Maytenus canariensis* and *Withania aristata* were collected in Tenerife (Canarias), while fresh leaves of *Salvia canariensis* were collected in Gran Canaria (Canarias). In addition, fresh leaves of *Laurus azorica* and fresh stems and leaves of *Hedychium gardnerianum* were collected in São Miguel (Azores). Voucher specimens from plants collected in the Canary Islands were deposited in the University of La Laguna herbarium (SEGAI), whereas vouchers from plants collected in the Azores were deposited in Ruy Teles Palhinha herbarium (AZB) at the University of Azores (Table 6), after botanical classification by the taxonomist from each herbarium.

The plant material was brought to the partnered laboratory in each respective geographic location, cut into small pieces and dried for a week in the dark at room temperature, with the aid of a dehumidifier, being milled afterwards and the obtained powder stored till needed for extraction.

### 4.2. Preparation of Extracts and Fractions Thereof

A sample of dried material of each plant was extracted by maceration for 72 h, at a ratio of 1:10 (*w*/*v*) using ethanol at 96% as solvent, renewed every 24 h. The extract was filtered, and the solvent was removed to dryness on a rotary evaporator at 40 °C, yielding the residue of each ethanolic extract.

The dried ethanolic extract of *H. gardnerianum* was further suspended in water and fractionated by liquid–liquid partition, through the modified Kupchan method, with *n*-hexane and ethyl acetate, originating two organic fractions (HF and EAF) and one water residue (WF).

### 4.3. Insecticidal Activity

#### 4.3.1. Insect Rearing and Treatment

In this study, *C. capitata* specimens were obtained from a colony maintained for over 20 generations under laboratory conditions with a photoperiod of 14 h of light/10 h of dark, temperature of 24 ± 1 °C and humidity of 70 ± 5%. Larvae were tended with an artificial diet, according to the methodology described by Albajes and Santiago-Álvarez [42], and adults were supplemented with a mixture of water and yeast hydrolysate (FLUKA Analytical, Alcobendas, Spain) and sugar (1:3 *w*/*w*).

#### 4.3.2. Oviposition and Adults’ Mortality Assay

Stock solutions of the extracts and fractions were dissolved in ethanol for the assays at a concentration of 50 mg/mL. Each sample (ethanolic extracts or fractions) was evaluated regarding its effect on the mortality and oviposition of the fruit fly *C. capitata* using artificial fruits made from agar. This protocol was adapted and improved based on the work of Salles [43] and Furtado et al. [15].

Briefly, the artificial fruits were prepared from a solution containing 350 mL of distilled water, 75 mL of natural orange juice, 8.0 g of agar and 4 mL of methyl hydroxybenzoate solution (10% in ethanol), that was poured into half-sphere forms (*v* = 11 mL each). After cooling, the artificial fruits were wrapped in parafilm (used to mimic a natural fruit texture and to allow the samples to be spread more evenly over the surface) and put in plastic cups (8 cm of nozzle diameter by 10.5 cm of height), and 150 µL of 10% honey solution was applied on top of each artificial fruit and a brush used to cover all of the fruit’s surface, leaving to dry for one hour. Subsequently, 250 µL of the different samples dissolved in ethanol at a concentration of 50 mg/mL was added with a micropipette to the top of the artificial fruits and carefully spread with a brush, to ensure an even coating of the liquid over the fruit surface. The active principle of neem oil, azadirachtin, was purchased from Sigma-Aldrich (Aldrich chemical Co., St. Louis, MO, USA) at approximately 95% purity, and used as positive control at the recommended concentration of 10 µg/cm^2^ (the equivalent to brushing one artificial fruit with 250 µL of the solution at 0.88 mg/mL). After being left to dry for 2 to 4 h, 13 flies (3 males and 10 females) were selected and added to the cup. Mortality was checked every 24 h. After 72 h, mortality was checked and the artificial fruits were removed from the cups, to allow the number of eggs laid in each one to be counted. Each treatment was replicated ten times.

The percentage mortality was corrected by Abbott’s formula [44] whenever the control mortality was more than 5%. The numbers of eggs laid for the treated and control groups were used to determined oviposition deterrent activity using the formula [45]

$$\%OD=\frac{Cs-Ts}{Cs}\times 100$$

where:

*OD* = oviposition deterrent activity;
*Cs* = number of eggs laid on the control;
*Ts* = number of eggs laid in the treated container.

#### 4.3.3. Statistical Analysis

All data were analysed using a General Linear Model (GLM), and means were separated using the Least Significant Difference (LSD) test. For all analyses, SPSS Statistics version 26.0 (IBM, Armonk, NY, USA) was used. The percentage mortality was corrected by Abbott’s formula [44] whenever the control mortality was above 5%. Time–response data obtained by the contact toxicity assay were submitted to probit analysis (IBM SPSS statistics) to determine lethal times (LT_50_ and LT_90_), at a single concentration.

### 4.4. Phytochemical Analysis

The chemical composition of the water fraction of *H. gardnerianum*, the sample with the best activity against *C. capitata*, was analyzed by HPLC-PDA-MS/MS. The dried fraction (7.58 mg) was dissolved in 1.5 mL of MeOH/H_2_O mixture (40%), filtered and injected into the HPLC system.

Chromatographic separation was carried out on an Agilent 1200 Infinity system (Agilent Technologies, Santa Clara, CA) equipped with an Agilent Poroshell 120 EC-C18 column (100 × 4.6 mm i.d.) with 2.7 µm particle size, operating at a temperature of 40 °C. A binary mobile phase with a gradient elution was used. Mobile phase A was H_2_O with 0.1% of formic acid, and mobile phase B was CH_3_CN with 0.1% formic acid. The flow rate was set for 0.6 mL/min. The elution gradient was as follows: 0% B to 40% B in 20 min, 90% B in 5 min and then 5 min at 90% B; afterwards the analytical column was re-equilibrated to initial gradient settings. The sample injection volume was 5 µL.

MSD Trap XCT mass spectrometer (Agilent Technologies, Santa Clara, CA, USA) with an ESI- ion source was used for the experiments. The following mass spectrometer parameters were applied: capillary voltage +3500 V; nebulizer (N_2_)—60 psi; drying gas (N_2_) temperature and flowrate—350 °C and 12 L/min, respectively; trap accumulation time—200 ms; trap Smart Target value—50,000; mass range 100 to 1500 *m*/*z*. MS/MS experiments were applied for the identification of compounds using helium as collision gas. Data analysis was performed using Agilent ChemStation software (A.10.02). The rutin trihydrate (CAS-Nr. 250249-75-3) was obtained from Alfa Aesar (Karlsruhe, Germany) at 97%, and was used as standard to identify compound **5** (Table 5).

## 5. Conclusions

The results presented above support the notion that plant-derived extracts are promising sources of biopesticides for application in integrated pest management against *C. capitata*. The possibility of using ethanolic extracts obtained by maceration at room temperature for this purpose is particularly interesting due to the low environmental impact and simplicity of this type of preparation. It can be carried out without specialized equipment and be easily applied by farmers.

Considering the information available in the literature [17], this is the first time that ethanolic extracts of *A. frutescens*, *C. symphytifolius*, *H. gardnerianum*, *L. azorica*, *M. canariensis*, *S. canariensis* and *W. aristata* have been tested against *C. capitata*. Furthermore, these findings also confirm the potential of *H. gardnerianum* as the best candidate, among the plants tested, to be used against *C. capitata*. Additionally, the liquid–liquid partition led to an improvement in the bioactivity of some fractions, with the most polar fraction being the one that showed the greatest activity against *C. capitata*. In fact, the water fraction from *H. gardnerianum* exhibited a mortality rate (57.69%) and oviposition deterrent activity (83.43%) greater than the original ethanolic extract (21.15% and 78.30%, respectively), both at 50 mg/mL, and higher than the values obtained with the commercial pesticide azadirachtin (25.93% and 35.25%) at the recommended concentration of 0.88 mg/mL. The activity of the water fraction can be attributed to its chemical composition, containing triterpene derivatives and mainly glycosides of myricetin and quercetin, including rutin, described in the literature as a biopesticide [31].

Although very promising, further tests must be carried out concerning the effect of these extracts on auxiliary and pollinating insects to assess their environmental safety. As a final remark, it should be highlighted that due to the current lack of cost-effective and easily implementable tools for *C. capitata* management, this topic holds significance, and this paper aims to contribute as another step toward finding a solution for this problem. Future studies should also focus on determining the mechanism of action of the observed biopesticide effect. 

## Figures and Tables

**Table 1:** Ethanolic extract yield (% dry weight).

Plants	Ethanolic Extract Yield
*Argyranthemum frutescens*	4.81
*Cistus symphytifolius*	14.13
*Hedychium gardnerianum*	6.89
*Laurus azorica*	13.21
*Maytenus canariensis*	14.77
*Salvia canariensis*	13.41
*Withania aristata*	12.68

**Table 2:** Mortality rates (mean ± SEM) with Abbott’s correction of *Ceratitis capitata* after contact with plant extracts.

Plants (Extracts and Fractions)		Adults’ Mortality (% at 50 mg/mL) ^§^		
		24 h ^#^	48 h	72 h
*Argyranthemum frutescens* EE		2.82 ± 1.61	5.11 ± 4.17 a	14.07 ± 7.87 abc
*Cistus symphytifolius* EE		4.93 ± 3.48	7.30 ± 4.89 a	22.96 ± 5.42 abc
*Hedychium gardnerianum* EE		−0.86 ± 0.00	9.82 ± 7.49 a	21.15 ± 14.64 abc
*Laurus azorica* EE		−0.86 ± 0.00	−0.89 ± 1.85 a	1.92 ± 4.74 ab
*Maytenus canariensis* EE		2.11 ± 1.18	2.92 ± 2.53 a	14.07 ± 6.81 abc
*Salvia canariensis* EE		0.70 ± 1.41	7.30 ± 4.89 a	27.41 ± 11.39 bc
*Withania aristata* EE		1.41 ± 1.09	7.30 ± 3.49 a	20.00 ± 9.69 abc
*Hedychium gardnerianum* EE	HF	0.07 ± 0.89	11.46 ± 8.85 a	32.69 ± 14.01 cdf
	EAF	−0.86 ± 0.00	0.89 ± 2.54 a	−1.92 ± 5.26 a
	WF	5.17 ± 4.03	31.25 ± 11.16 b	57.69 ± 12.35 d
Azadirachtin *		6.34 ± 2.85	13.14 ± 6.01 a	25.93 ± 7.24 bef
Control		0.00 ± 0.82	0.00 ± 2.24 a	0.00 ± 4.95 ae
*F*		1.717	2.704	2.739
df		11	11	11
*p*		0.079	0.028	0.004

**^§^** Each value is based on ten replicas. * Azadirachtin used as a positive control at recommended concentration 10 µg/cm^2^ (0.88 mg/mL). ^#^ The differences in all values presented in this column are not statistically significant. GLM *F*, df and *p*-values. Mean values in each column followed by a different letter are significantly different, based on GLM test followed by LSD test (*p* < 0.05). EE—ethanolic extract; HF—hexane fraction; EAF—ethyl acetate fraction; WF—water fraction.

**Table 3:** Lethal time (LT_50_ and LT_90_) of *Hedychium gardnerianum* fractions against adults of *Ceratitis capitata* after 72 h exposure.

Samples	N *	LT_50_ ^1^	LT_90_ ^1^	Slope (±SEM)	Intercept (±SEM)	H ^2^
Hexane fraction of *H. gardnerianum* EE	116	95.29 (81.92–129.65)	182.40 (132.87–366.01)	4.55 ± 0.88	−9.00 ± 1.58	0.46
Water fraction of *H. gardnerianum* EE	117	72.54 (64.62–85.71)	167.5 (128.89–260.79)	3.53 ± 0.48	−6.60 ± 0.83	0.01

* Number of insects tested. ^1^ LT_50_ and LT_90_ expressed as the number of hours required to kill 50 and 90% of insect adult population, respectively. The differences in the values LT_50_ and LT_90_ are not significantly different based on non-overlapping 95% confidence limit. ^2^ H—Heterogeneity factor (χ2/df).

**Table 4:** Oviposition deterrent activity (OD %) of *Ceratitis capitata* after contact with plant extracts.

Plants (Extracts and Fractions)		% OD (mean ± SEM) ^§^
*Argyranthemum frutescens* EE		69.83 ± 6.25 c
*Cistus symphytifolius* EE		47.46 ± 6.51 bc
*Hedychium gardnerianum* EE		78.30 ± 7.06 c
*Laurus azorica* EE		55.11 ± 12.22 bc
*Maytenus canariensis* EE		7.46 ± 26.53 ab
*Salvia canariensis* EE		38.64 ± 13.04 bc
*Withania aristata* EE		−44.41 ± 43.30 a
*Hedychium gardnerianum* EE	HF	78.70 ± 7.81 c
	EAF	35.51 ± 25.55 bc
	WF	83.43 ± 6.22 c
Azadirachtin		35.25 ± 42.78 bc
Control		0.00 ± 21.21 ab
*F*		3.343
df		11
*p*		0.001

**^§^** Each value is based on ten replicas. GLM *F*, df and *p*-values. Mean values in each column followed by a different letter are significantly different, based on GLM test followed by LSD test (*p* <0.05).

**Table 5:** HPLC-MS/MS analysis of the major compounds in the water fraction (WF) of the *H. gardnerianum* ethanol extract.

Peak Number	Retention Time (min)	[M-H]^−^ (*m*/*z*)	MS/MS Ions (*m*/*z*)	Assigned Compound
**1**	10.7	625	479; 317	Myricetin-3-*O*-rhamnosyl-glucoside
**2**	11.8	609	463; 447; 301	Quercetin-3-*O*-glucoside-7-*O*-rhamnoside
**3**	12.2	925	779; 633; 597	Triterpene-3-*O*-di-rhamnoside
**4**	13.4	625	317; 271; 179	Myricetin-3-*O*-rutinoside
**5**	14.5	609	301; 179	Quercetin-3-*O*-rutinoside ^1^
**6**	14.9	625	463; 317	Myricetin-3-*O*-glucosyl-rhamnoside
**7**	15.1	477	301; 179	Quercetin-3-*O*-glucuronide
**8**	18.8	685	639; 621	Triterpene derivative
**9**	19.4	821	785; 767; 755; 737; 725	Triterpene derivative

^1^ Identification based on the comparation of rutin standard injection.

**Table 6:** Details of collected plant species under study.

Plant Species ^1^	Plant Part	Voucher Code
*Argyranthemum frutescens* (L.) Sch.Bip.	Leaves	TFC 54.141 ^2^
*Cistus symphytifolius* Lam.	Leaves	TFC 53.703 ^2^
*Gymnosporia cassinoides* (L’Hér.) Masf. (syn. *Maytenus canariensis* (Loes.) G.Kunkel & Sunding)	Leaves	TFC 53.244 ^2^
*Hedychium gardnerianum* Sheppard ex Ker Gawl.	Leaves and Stems	3671 ^3^
*Laurus azorica* (Seub.) Franco	Leaves	3670 ^3^
*Salvia canariensis* L.	Leaves	TFC 53.328 ^2^
*Withania aristata* (Aiton) Pauquy	Leaves	TFC 53.219 ^2^

^1^ POWO (2023). Plants of the World Online—Royal Botanic Gardens, Kew. https://powo.science.kew.org/, accessed on 27 October 2023. ^2^ Voucher specimens deposited at the herbarium TFC (SEGAI) Universidad de La Laguna, Tenerife, Spain. ^3^ Voucher specimens deposited at the Ruy Teles Palhinha herbarium (AZB), University of Azores, Ponta Delgada, Azores, Portugal.

## Data Availability

The data presented on this study are available in the article.

## References

[1] Sciarretta A., Tabilio M.R., Lampazzi E., Ceccaroli C., Colacci M., Trematerra P. (2018). Analysis of the Mediterranean fruit fly [*Ceratitis capitata* (Wiedemann)] spatio-temporal distribution in relation to sex and female mating status for precision IPM. PLoS ONE.

[2] Ruiz-Arce R., Todd T.N., Deleon R., Barr N.B., Virgilio M., De Meyer M., McPheron B.A. (2020). Worldwide phylogeography of *Ceratitis capitata* (Diptera: Tephritidae) using mitochondrial DNA. J. Econ. Entomol..

[3] Deschepper P., Todd T.N., Virgilio M., De Meyer M., Barr N.B., Ruiz-Arce R. (2021). Looking at the big picture: Worldwide population structure and range expansion of the cosmopolitan pest *Ceratitis capitata* (Diptera, Tephritidae). Biol. Invasions.

[4] Arias M.B., Hartle-Mougiou K., Taboada S., Vogler A.P., Riesgo A., Elfekih S. (2022). Unveiling biogeographical patterns in the worldwide distributed *Ceratitis capitata* (medfly) using population genomics and microbiome composition. Mol. Ecol..

[5] Ordax M., Piquer-Salcedo J.E., Santander R.D., Sabater-Muñoz B., Biosca E.G., López M.M., Marco-Noales E. (2015). Medfly *Ceratitis capitata* as potential vector for fire blight pathogen *Erwinia amylovora*: Survival and transmission. PLoS ONE.

[6] Zaada D.S.Y., Ben-Yosef M., Yuval B., Jurkevitch E. (2019). The host fruit amplifies mutualistic interaction between *Ceratitis capitata* larvae and associated bacteria. BMC Biotechnol..

[7] Magaña C., Hernández-Crespo P., Brun-Barale A., Couso-Ferrer F., Bride J.-M., Castañera P., Feyereisen R., Ortego F. (2008). Mechanisms of resistance to malathion in the medfly *Ceratitis capitata*. Insect Biochem. Mol. Biol..

[8] Badr A.M. (2020). Organophosphate toxicity: Updates of malathion potential toxic effects in mammals and potential treatments. Environ. Sci. Pollut. Res..

[9] Khursheed A., Rather M.A., Jain V., Wani A.R., Rasool S., Nazir R., Malik N.A., Majid S.A. (2022). Plant based natural products as potential ecofriendly and safer biopesticides: A comprehensive overview of their advantages over conventional pesticides, limitations and regulatory aspects. Microb. Pathog..

[10] Grzywacz D., Stevenson P.C., Mushobozi W.L., Belmain S., Wilson K. (2014). The use of indigenous ecological resources for pest control in Africa. Food Sec..

[11] Stevenson P.C., Belmain S.R. (2016). Pesticidal plants in African agriculture: Local uses and global perspectives. Outlooks Pest. Manag..

[12] Mobolade A.J., Bunindro N., Sahoo D., Rajashekar Y. (2019). Traditional methods of food grains preservation and storage in Nigeria and India. Ann. Agric. Sci..

[13] Gonzalez-Coloma A., Reina M., Diaz C.E., Fraga B.M., Santana-Meridas O. (2013). Natural product-based biopesticides for insect control. Elsevier Reference Module in Chemistry, Molecular Sciences and Chemical Engineering.

[14] López S.B., López M.L., Aragón L.M., Tereschuk M.L., Slanis A.C., Feresin G.E., Zygadlo J.A., Tapia A.A. (2011). Composition and anti-insect activity of essential oils from *Tagetes* L. species (Asteraceae, Helenieae) on *Ceratitis capitata* Wiedemann and *Triatoma infestans* Klug. J. Agric. Food Chem..

[15] Furtado R., Baptista J., Lima E., Paiva L., Barroso J.G., Rosa J.S., Oliveira L. (2014). Chemical composition and biological activities of *Laurus* essential oils from different Macaronesian Islands. Biochem. Syst. Ecol..

[16] Ghalbane I., Alahyane H., Aboussaid H., Chouikh N.-E., Costa J., Romane A., El Messoussi S. (2022). Chemical composition and insecticidal properties of Moroccan *Lavandula dentata* and *Lavandula stoechas* essential oils against Mediterranean fruit fly, *Ceratitis capitata*. Neotrop. Entomol..

[17] Tavares W.R., Barreto M.d.C., Seca A.M.L. (2021). Aqueous and ethanolic plant extracts as bio-insecticides—Establishing a bridge between raw scientific data and practical reality. Plants.

[18] Ghabbari M., Guarino S., Caleca V., Saiano F., Sinacori M., Baser N., Jemâa J.M.-B., Lo Verde G. (2018). Behavior-modifying and insecticidal effects of plant extracts on adults of *Ceratitis capitata* (Wiedemann) (Diptera Tephritidae). J. Pest Sci..

[19] Stupp P., Rakes M., Martins L.N., Piovesan B., Oliveira D.C., Miranda J.A.C., Ribeiro L.P., Nava D.E., Bernardi D. (2020). Lethal and sublethal toxicities of acetogenin-based bioinsecticides on *Ceratitis capitata* and the parasitoid *Diachasmimorpha longicaudata*. Phytoparasitica.

[20] Kumar S., Singh A., Kumar B. (2017). Identification and characterization of phenolics and terpenoids from ethanolic extracts of *Phyllanthus* species by HPLC-ESI-QTOF-MS/MS. J. Pharm. Anal..

[21] Ablajan K., Abliz Z., Shang X.-Y., He J.-M., Zhang R.-P., Shi J.-G. (2006). Structural characterization of flavonol 3,7-di-*O*-glycosides and determination of the glycosylation position by using negative ion electrospray ionization tandem mass spectrometry. J. Mass Spectrom..

[22] Cuyckens F., Claeys M. (2004). Mass spectrometry in the structural analysis of flavonoids. J. Mass Spectrom..

[23] Singh A., Kumar S., Bajpai V., Reddy T.J., Rameshkumar K.B., Kumar B. (2015). Structural characterization of flavonoid *C*- and *O*-glycosides in an extract of *Adhatoda vasica* leaves by liquid chromatography with quadrupole time-of-flight mass spectrometry. Rapid Commun. Mass Spectrom..

[24] Roepke J., Bozzo G.G. (2013). Biocatalytic synthesis of quercetin 3-*O*-glucoside-7-*O*-rhamnoside by metabolic engineering of *Escherichia coli*. Chembiochem..

[25] Singh A.P., Chitme H., Sharma R.K., Kandpal J.B., Behera A., Abdel-Wahab B.A., Orabi M.A., Khateeb M.M., Habeeb M.S., Bakir M.B. (2023). A comprehensive review on pharmacologically active phyto-constituents from *Hedychium* species. Molecules.

[26] Sobral F., Calhelha R.C., Barros L., Dueñas M., Tomás A., Santos-Buelga C., Vilas-Boas M., Ferreira I.C.F.R. (2017). Flavonoid composition and antitumor activity of bee bread collected in Northeast Portugal. Molecules.

[27] Guillem-Amat A., Sánchez L., López-Errasquín E., Ureña E., Hernández-Crespo P., Ortego F. (2020). Field detection and predicted evolution of spinosad resistance in *Ceratitis capitata*. Pest Manag. Sci..

[28] Williams C.A., Harborne J.B. (1977). The leaf flavonoids of the Zingiberales. Biochem. Syst. Ecol..

[29] Akhov L., Barl B. (2003). Isolation of quercetin glycosides from leaves of sea buckthorn (*Hippophae rhamnoides* ssp. *mongolica*). Acta Hortic..

[30] Guimarães R., Barros L., Dueñas M., Carvalho A.M., Queiroz M.J.R.P., Santos-Buelga C., Ferreira I.C.F.R. (2013). Characterization of phenolic compounds in wild fruits from Northeastern Portugal. Food Chem..

[31] Jadhav D.R., Mallikarjuna N., Rathore A., Pokle D. (2012). Effect of some flavonoids on survival and development of *Helicoverpa armigera* (Hübner) and *Spodoptera litura* (Fab) (Lepidoptera: Noctuidae). Asian J. Agric. Sci..

[32] Mitchell M.J., Keogh D.P., Crooks J.R., Smith S.L. (1993). Effects of plant flavonoids and other allelochemicals on insect cytochrome P-450 dependent steroid hydroxylase activity. Insect Biochem. Mol. Biol..

[33] Shamkh I.M., Al-Majidi M., Shntaif A.H., Kai P.T.D., Nh-Pham N., Rahman I., Hamza D., Khan M.S., Elsharayidi M.S., Salah E.T. (2022). Nontoxic and naturally occurring active compounds as potential inhibitors of biological targets in *Liriomyza trifolii*. Int. J. Mol. Sci..

[34] Jaleel W., Wang D., Lei Y., Qi G., Chen T., Rizvi S.A.H., Sethuraman V., He Y., Lu L. (2020). Evaluating the repellent effect of four botanicals against two *Bactrocera* species on mangoes. PeerJ.

[35] Sharifi M., Ghadamyari M., Sajedi R.H., Mahmoodi N.O. (2015). Effects of 4-hexylresorcinol on the phenoloxidase from *Hyphantria cunea* (Lepidoptera: Arctiidae): *In vivo* and *in vitro* studies. Insect Sci..

[36] Scott I.M., Samara R., Renaud J.B., Sumarah M.W. (2017). Plant growth regulator-mediated anti-herbivore responses of cabbage (*Brassica oleracea*) against cabbage looper *Trichoplusia ni* Hübner (Lepidoptera: Noctuidae). Pestic. Biochem. Physiol..

[37] Cui B., Huang X., Li S., Hao K., Chang B.H., Tu X., Pang B., Zhang Z. (2019). Quercetin affects the growth and development of the grasshopper *Oedaleus asiaticus* (Orthoptera: Acrididae). J. Econ. Entomol..

[38] De Geyter E., Swevers L., Caccia S., Geelen D., Smagghe G. (2012). Saponins show high entomotoxicity by cell membrane permeation in Lepidoptera. Pest Manag. Sci..

[39] Lin M., Yang S., Huang J., Zhou L. (2021). Insecticidal triterpenes in Meliaceae: Plant species, molecules and activities: Part I (*Aphanamixis-Chukrasia*). Int. J. Mol. Sci..

[40] Lin M., Bi X., Zhou L., Huang J. (2022). Insecticidal triterpenes in Meliaceae: Plant species, molecules and activities: Part II (*Cipadessa, Melia*). Int. J. Mol. Sci..

[41] Ma Y.F., Xiao C. (2013). Push-pull effects of three plant secondary metabolites on oviposition of the potato tuber moth, *Phthorimaea operculella*. J. Insect Sci..

[42] Albajes R., Santiago-Álvarez C. (1980). Efectos de la densidad larvaria y de la alimentación en la proporción de sexos de *Ceratitis capitata* (Diptera: Tephritidae). Anales INIA Serie Agrícola.

[43] Salles L.A.B. (1992). Metodologia de criação de *Anastrepha fraterculus* (Wied., 1830) (Diptera: Ttephritidae) em dieta artificial em laboratório. ASEB.

[44] Abbott W.S. (1925). A method of computing the effectiveness of an insecticide. J. Econ. Entomol..

[45] Hematpoor A., Liew S.Y., Azirun M.S., Awang K. (2017). Insecticidal activity and the mechanism of action of three phenylpropanoids isolated from the roots of *Piper sarmentosum* Roxb. Sci. Rep..

